# Coffee and Its Major Polyphenols in the Prevention and Management of Type 2 Diabetes: A Comprehensive Review

**DOI:** 10.3390/ijms26125544

**Published:** 2025-06-10

**Authors:** HwiCheol Kim, Sang Ryong Kim, Un Ju Jung

**Affiliations:** 1 Department of Food Science and Nutrition, Pukyong National University, 45 Yongso-ro, Nam-gu, Busan 48513, Republic of Korea; k423897@naver.com; 2 BK21 FOUR KNU Creative BioResearch Group, School of Life Science and Biotechnology, Kyungpook National University, 80 Daehak-ro, Buk-gu, Daegu 41566, Republic of Korea

**Keywords:** type 2 diabetes mellitus, coffee, polyphenols, chlorogenic acid, caffeic acid, ferulic acid, *p*-coumaric acid, sinapic acid

## Abstract

Type 2 diabetes mellitus (T2DM) is a chronic metabolic disorder characterized by insulin resistance and impaired glucose metabolism and affects a substantial portion of the global population. Over the past few decades, numerous studies have investigated lifestyle factors, including diet and physical activity, as preventive measures or adjunctive treatments for T2DM. Among the dietary factors, coffee consumption has garnered attention because of its potential to mitigate the risk and progression of T2DM. This review examines the current evidence on the relationship between coffee consumption and T2DM, with particular focus on the major polyphenols found in coffee, such as chlorogenic acid and related hydroxycinnamic acids (caffeic acid, ferulic acid, *p*-coumaric acid, and sinapic acid). These bioactive compounds are thought to exert anti-diabetic effects through several mechanisms, including improvements in glucose homeostasis, insulin sensitivity, inflammation, and oxidative stress. This review aimed to clarify the scientific rationale behind the potential therapeutic effects of coffee on T2DM and proposed directions for future studies. However, significant knowledge gaps remain, including limited clinical evidence, unclear optimal dosages, low bioavailability, and an incomplete understanding of molecular mechanisms. Addressing these gaps through well-designed clinical trials and advanced molecular studies is essential to fully establish the therapeutic potential of coffee and its polyphenols in T2DM.

## 1. Introduction

Type 2 diabetes mellitus (T2DM) is a serious and growing global health challenge, and its prevalence is increasing at an alarming rate. The World Health Organization reported that the number of adults with diabetes aged ≥ 18 years increased from 7% (200 million) in 1990 to 14% (830 million) in 2022, with its incidence continuing to rise because of aging populations, urbanization, and lifestyle changes [1]. The International Diabetes Federation estimated that 537 million adults aged 20–79 years had diabetes by 2021 based on epidemiological data that encompassed both diagnosed and undiagnosed cases [2]. This number is projected to reach 783 million by 2045, if current trends continue. Although these estimates differ in methodology, both organizations highlight the alarming growth of diabetes incidence worldwide and its significant impact on public health. T2DM accounts for approximately 90% of all diabetes cases globally [2].

T2DM is a complex metabolic disorder characterized by insulin resistance and impaired glucose homeostasis, often leading to severe complications such as cardiovascular disease, nephropathy, and neuropathy [3]. Lifestyle modifications, including dietary interventions and regular physical activity, play crucial roles in maintaining blood glucose control and preventing complications in T2DM [4]. Although some individuals may achieve glycemic control through lifestyle changes alone, pharmacological treatments are often necessary, especially in more advanced or severe cases, to prevent disease progression and optimize long-term health outcomes. Pharmacological treatments such as metformin, sodium glucose cotransporter 2 (SGLT2) inhibitors, and glucagon-like peptide-1 (GLP-1) receptor agonists are generally effective and well-tolerated, and have significantly contributed to improving glycemic control and preventing complications in T2DM [5]. However, they may be associated with certain side effects, such as gastrointestinal side effects, ketoacidosis, and the risk of hypoglycemia [5]. Therefore, there is an increasing need for safer and more sustainable therapies. A wide range of bioactive compounds, including those derived from plants, microalgae, and insects, are being explored for their potential in metabolic disease management [6,7,8]. Plant-derived compounds have gained attention as potential therapeutic agents because of their broad bioactive properties and lower risk of adverse effects compared to synthetic drugs [8], although occasional adverse effects have been reported [9]. Polyphenol-rich plant foods and beverages have positive metabolic and mental health benefits [10,11]. Many phytochemicals exhibit antioxidant, anti-inflammatory, and insulin-sensitizing effects, making them promising candidates for diabetes management [8]. Coffee and its bioactive compounds have been extensively studied for their potential role in modulating glucose metabolism and reducing the risk of T2DM and its complications [12,13].

Coffee is one of the most widely consumed beverages worldwide [14]. Global coffee consumption has increased by approximately 7.8% over the past five years, reaching more than 10 million tons in 2022/23 [14]. Moreover, many studies suggest that coffee may help in mitigating the risk of developing T2DM [12,13]. In fact, epidemiological studies have reported that regular coffee drinkers exhibit a significantly lower risk of developing T2DM than non-drinkers. This protective effect is believed to be due to the presence of various bioactive compounds in coffee, such as chlorogenic acid and related hydroxycinnamic acids (namely, caffeic, ferulic, *p*-coumaric, and sinapic acids). These bioactive compounds may influence various mechanisms involved in glucose regulation, insulin resistance, inflammation, and oxidative stress, all of which are critical factors in the pathogenesis of T2DM.

This review aimed to provide a comprehensive overview of the relationship between coffee consumption and T2DM, with a particular focus on the major polyphenols found in coffee, such as chlorogenic acid and its structurally related hydroxycinnamic acids (caffeic, ferulic, *p*-coumaric, and sinapic acids), which influence key pathways involved in glucose homeostasis, insulin resistance, oxidative stress, and inflammation. It also discusses the current research gaps and proposes future directions for clinical investigations.

## 2. Coffee and T2DM

### 2.1. Evidence from Epidemiological and Clinical Studies

Numerous epidemiological and clinical studies have investigated the relationship between coffee consumption and the incidence and progression of T2DM in humans. Overall, evidence from prospective cohort studies and meta-analyses strongly supports an inverse association between habitual coffee intake and T2DM risk [15,16,17,18,19,20]. Notably, beneficial effects were observed for both caffeinated and decaffeinated coffees [15,19].

Large-scale evidence from meta-analyses and prospective cohort studies consistently indicates the association between higher coffee consumption and a significantly lower risk of T2DM development, as compared with minimal or no coffee intake. For instance, a meta-analysis by Huxley et al. [15], which included over 457,000 participants aged 20 to 98 years from 18 prospective cohort studies (median follow-up duration: 2–20 years), showed a dose-dependent inverse association, with each additional daily cup of coffee reducing the risk of T2DM by 7% (relative risk [RR]: 0.93; 95% confidence interval [CI]: 0.91–0.95). Similar protective effects were also observed for decaffeinated coffee, suggesting a role for non-caffeinated bioactive compounds [15]. These findings are supported by U.S. cohort studies. Bhupathiraju et al. [16] analyzed over 120,000 participants aged 30 to 55 years across three major U.S. cohorts and reported that increasing coffee intake by more than one cup per day over a 4-year period was associated with a 12% lower risk of T2DM in the subsequent 4 years. Conversely, reduced coffee intake was associated with higher risk, indicating the importance of habitual consumption patterns. Similar trends have been reported in other Asian populations. Iso et al. [17] found that Japanese adults aged 40 to 65 years with no history of T2DM who consumed ≥ 3 cups of coffee daily had a 42% lower risk of T2DM over a 5-year follow-up compared to those consuming < 1 cup per week (odds ratio: 0.58; 95% CI: 0.37–0.90), with stronger effects noted in women and overweight individuals.

A systematic review of 13 cohort studies published between 2001 and 2011 confirmed an inverse association between habitual coffee consumption and the risk of T2DM [18]. Among over 1.2 million participants aged 20–88 years and 9473 cases of incident T2DM, those consuming 4–6 or more than 6–7 cups per day had significantly lower risks than those consuming < 2 cups daily, with follow-up durations ranging from 5 to 18 years. Each of the 13 cohort studies included in this systematic review reported odds ratios and confidence intervals [18]. This review also suggests that filtered and decaffeinated coffee may be more beneficial than boiled or caffeinated coffee, particularly in individuals aged < 60 years. However, the authors cautioned against promoting coffee consumption as a public health strategy without further mechanistic evidence. Potential adverse effects associated with high coffee intake should be carefully considered. Excessive consumption (more than 4 cups per day) has been linked to increased blood pressure in individuals with hypertension and reduced fertility in both men and women [21]. Caffeine, a major component of coffee, has also been associated with symptoms of nervousness, anxiety, depression, and an increased need for anxiolytic medications [21].

Recent meta-analytical evidence further strengthens the protective role of coffee. Ding et al. [19] conducted an updated meta-analysis of 28 prospective cohort studies involving over 1.1 million participants aged 20–88 years and 45,335 patients with T2DM, with follow-up durations ranging from 10 months to 20 years. They found a clear dose–response association (RR for 6 cups/day: 0.67; 95% CI: 0.61–0.74), with both caffeinated and decaffeinated coffee offering protection (RR per 1 cup/day: 0.91 and 0.94, respectively; *p* = 0.17). Jiang et al. [20] further confirmed these findings in another large-scale meta-analysis including over one million participants and 50,000 T2DM cases, with varied follow-up durations ranging from 2.6 to 24 years. They reported 29%, 21%, and 30% lower risks associated with coffee, decaffeinated coffee, and caffeine intake, respectively, when comparing the highest and lowest consumption groups. Notably, the associations were stronger among women, non-smokers, and those with a lower BMI (<25 kg/m^2^), implying that individual factors may modify the protective effects of coffee.

Despite the strong associations demonstrated across large observational cohorts, the question remains as to whether these relationships are causal. Several short-term randomized controlled trials (RCTs) have reported that caffeine intake can acutely reduce insulin sensitivity [22,23] and increase blood glucose concentrations [24,25]. However, as coffee contains numerous bioactive compounds in addition to caffeine, including chlorogenic acids, other polyphenols, and diterpenes, it is important to distinguish between the metabolic effects of whole coffee and those of caffeine alone. In this context, van Dam et al. [26] conducted two crossover trials in healthy individuals to assess the impact of regular coffee and isolated caffeine over 4 weeks. Regular coffee consumption significantly increased fasting insulin concentrations compared with coffee-free conditions, whereas caffeine and weaker coffee consumption showed a non-significant trend toward increased insulin levels. No significant changes in fasting glucose levels were observed. Although insulin resistance was not measured directly, an increase in fasting insulin levels following regular coffee consumption may reflect a short-term reduction in insulin sensitivity.

In addition to short-term findings, an 8-week RCT involving overweight but otherwise healthy adults found that both caffeinated and decaffeinated coffee altered metabolic biomarkers linked to adipose tissue and liver function [27]. Specifically, caffeinated coffee increased circulating adiponectin, a hormone that enhances insulin sensitivity [28], whereas decaffeinated coffee significantly reduced the levels of fetuin-A, a hepatokine as-sociated with insulin resistance [29]. Notably, no significant effects on glucose tolerance or insulin sensitivity were observed, suggesting that the metabolic benefits of coffee may arise from improvements in tissue function rather than from immediate effects on glycemia. These findings align with the results of another clinical study in habitual coffee drinkers, which further supports the role of non-caffeine components in mediating metabolic benefits through non-glycemic pathways [30]. In this trial, increasing coffee intake to eight cups per day over two months significantly elevated the serum concentrations of chlorogenic and caffeic acid metabolites, reduced inflammatory and oxidative stress markers such as interleukin (IL)-18 and 8-isoprostane, and increased the levels of adiponectin and high-density lipoprotein (HDL) cholesterol. Although glucose tolerance remained unchanged, these results highlight the potential of chlorogenic and caffeic acid metabolites in improving metabolic health through mechanisms other than glycemic regulation.

Further supporting the metabolic potential of chlorogenic acid, a clinical study demonstrated that the consumption of chlorogenic acid-enriched instant coffee reduced glucose absorption compared with control coffee in healthy individuals [31]. Notably, this effect was not observed in either regular or decaffeinated coffee, emphasizing the distinct role of chlorogenic acid. In a 12-week randomized double-blind trial involving overweight individuals, chlorogenic acid-enriched coffee led to significantly greater reductions in body weight [31], suggesting enhanced postprandial glucose regulation and weight loss associated with chlorogenic acid.

Long-term clinical trials have demonstrated the benefits of habitual coffee consumption on glucose metabolism. A 16-week randomized trial involving overweight men with impaired fasting glucose revealed that consumption of five cups of coffee per day alleviated glucose intolerance compared to that in non-coffee controls [32]. While decaffeinated coffee did not produce statistically significant changes in the results of the oral glucose tolerance test, consumption of both caffeinated and decaffeinated coffee was associated with improved post-load glucose after adjusting for waist circumference, implying that both caffeine and non-caffeine components may contribute to metabolic improvements.

Recent clinical evidence also suggests that the phenolic components of coffee, particularly polyphenols such as chlorogenic acid, may influence body composition. In a randomized, single-blind, crossover trial, overweight and obese adults consumed either lightly or heavily roasted coffee for 12 weeks [33]. Lightly roasted coffee, which contained higher levels of hydroxycinnamic acid (~400 mg/cup) than heavily roasted coffee, led to significantly greater reductions in fat mass and fat percentage. Both coffee types modestly increased muscle mass without altering body weight or metabolic syndrome markers (fasting blood glucose, HDL cholesterol, and triglycerides). These results highlight how differences in coffee preparation can influence the retention of bioactive compounds, and consequently, their metabolic effects.

Collectively, both caffeine and polyphenols in coffee contribute to improved insulin sensitivity, glucose metabolism, and inflammation modulation in individuals with T2DM, as supported by various observational and interventional studies. Beyond the diabetic population, some evidence from healthy individuals suggests that regular coffee consumption may also confer systemic benefits through antioxidative and rheological mechanisms. For instance, consumption of two cups of coffee per day for 3 weeks was shown to improve the glutathione redox ratio in erythrocytes and enhance blood rheology in healthy young adults, suggesting an additional mechanism by which coffee may exert health-promoting effects [34].

Table 1 summarizes the key epidemiological and clinical studies evaluating the relationship between coffee consumption and the risk of T2DM. A consistent inverse association has been observed across diverse populations and study designs, with both caffeinated and decaffeinated coffees exhibiting protective effects. Notably, chlorogenic acid-enriched or lightly roasted coffee, which retains high levels of polyphenols, demonstrates additional metabolic benefits, including reduced fat mass, improved glucose intolerance, and increased adiponectin levels.

### 2.2. Experimental Evidence from Animal Studies

Animal studies have provided compelling evidence supporting the beneficial metabolic effects of coffee, particularly in models of diet-induced insulin resistance and T2DM.

Chronic coffee consumption was shown to improve glucose tolerance, lower fasting blood glucose levels, and enhance insulin sensitivity in a high-fat-diet (HFD)-induced insulin resistance mouse model [35]. Moreover, coffee intake reduces systemic inflammation, as evidenced by decreased levels of pro-inflammatory cytokines such as tumor necrosis factor-α (TNF-α) and IL-6 [36,37,38]. In type 2 diabetic *KK-Ay* mice, coffee intake has been shown to protect against hyperglycemia by ameliorating insulin resistance, and these anti-diabetic effects are partly attributed to reduced inflammation in adipose tissue [37]. A recent study also reported that coffee consumption during HFD-induced obesity improved glucose intolerance in mice, which was accompanied by reductions in macrophage infiltration and expression of IL-6 and TNF-α in adipose tissue, as well as in plasma IL-6 levels [38].

The degree of coffee roasting may also influence physiological effects. A study in diet-induced obese rats compared the metabolic effects of unroasted, dark, and very dark-roasted coffee [39]. All three coffee types improved glucose tolerance and significantly lowered insulin levels and resistance, indicating enhanced insulin sensitivity. Notably, dark-roasted coffee lowered fasting glucose levels. Among the three types of coffee, unroasted coffee had the most pronounced lipid-lowering effects, including reductions in triglycerides, free fatty acids, and adipocyte size, all of which are closely linked to insulin resistance. Regardless of the degree of roasting, coffee intake attenuated hepatic steatosis and decreased the markers of hepatic apoptosis, suggesting a protective effect against liver dysfunction which is often associated with insulin resistance and metabolic dysregulation in T2DM [40].

In addition, animal studies have suggested that coffee reduces intestinal glucose absorption [41]. Although this effect may be negligible in healthy individuals with normal glucose metabolism, it may be beneficial in individuals with insulin resistance or diabetes by blunting postprandial glucose spikes. Whether this action is mediated by coffee-induced alterations in gut microbiota remains unclear and warrants further investigation.

Collectively, these animal data suggest the potential of coffee as a modulator of key molecular targets involved in glucose homeostasis, insulin activity, lipid metabolism, and hepatic function. The mechanistic insights gained from animal models contribute to a biologically plausible explanation for the protective associations observed between coffee consumption and T2DM risk in human studies. However, the differences in physiology, dosing, and study conditions between animal and human studies highlight the need for cautious interpretation and further validation in clinical settings.

## 3. Potential Role of Chlorogenic Acid and Related Hydroxycinnamic Acids in T2DM

Coffee contains a variety of bioactive compounds, including chlorogenic acids, caffeine, diterpenes, and trigonelline, many of which are widely recognized for their influence on diabetes. Along with caffeine, growing evidence suggests that non-caffeine compounds, such as chlorogenic acids, also play a significant role in glucose metabolism and insulin sensitivity.

**Figure 1:**
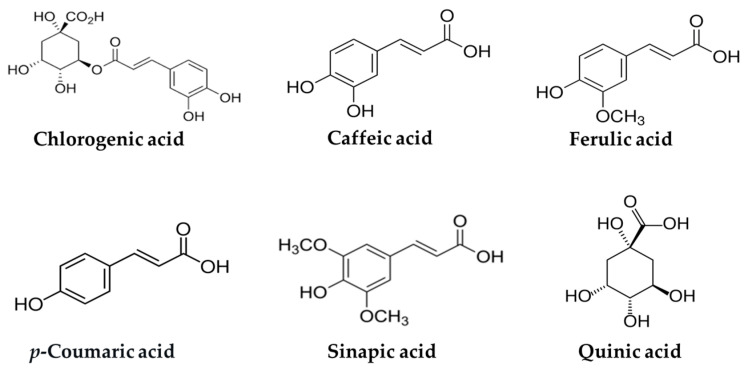
Chemical structures of chlorogenic acid and related hydroxycinnamic acids.

Chlorogenic acids are a family of polyphenolic compounds consisting of esters formed between hydroxycinnamic acids (caffeic, ferulic, *p*-coumaric, and sinapic acids) and quinic acid [42]. Among these, chlorogenic acid, an ester of caffeic and quinic acids, is the predominant form found in coffee. Figure 1 illustrates the chemical structures of chlorogenic, caffeic, ferulic, *p*-coumaric, sinapic, and quinic acids. The contents of chlorogenic acid and related hydroxycinnamic acids in coffee vary depending on the coffee species, roasting degree, and preparation method [43], and are summarized in Table 2.

**Figure 2:**
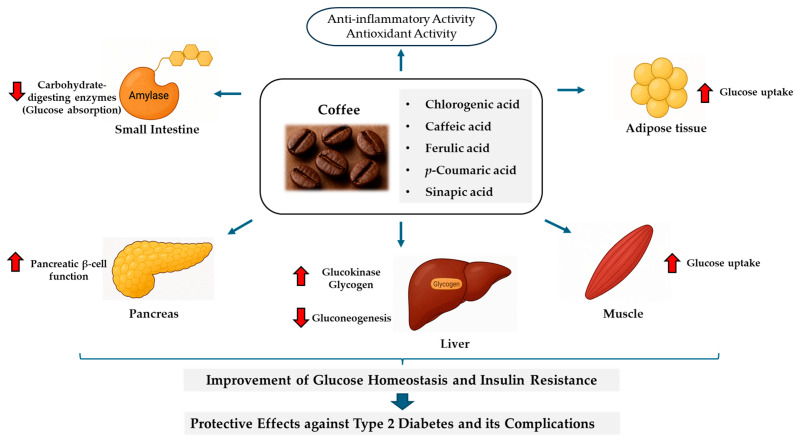
Potential anti-diabetic mechanisms of chlorogenic acid and related hydroxycinnamic acids. Red arrows indicate direction: ↑ increase, ↓ decrease.

By applying high-performance liquid chromatography (HPLC) following methanolic–acetic acid extraction and alkaline hydrolysis to coffee bean samples, Mattila et al. [44] characterized key phenolic acids (caffeic, *p*-coumaric, ferulic, sinapic), and Moeenfard et al. [45] quantified chlorogenic acid in coffee brews via HPLC with a diode array detector. These polyphenols of coffee, as characterized by HPLC in previous studies, may contribute to the anti-diabetic potential of coffee. Figure 2 presents a schematic overview of the potential anti-diabetic mechanisms of chlorogenic acid and related hydroxycinnamic acids, highlighting their roles in inhibiting carbohydrate-digesting enzymes, improving pancreatic β-cell function, regulating hepatic glucose metabolism, enhancing glucose uptake, and exerting antioxidant and anti-inflammatory effects. This figure provides a framework for the detailed discussion of mechanisms of each compound in the following sections.

**Table 2:** Contents of chlorogenic acid and related hydroxycinnamic acids in coffee.

Compound	Content in Coffee	Reference
Chlorogenic acid	76–84% of total chlorogenic acids; approx. 100 mg/g dry weight (green coffee beans)	[46]
Caffeic acid	63.1–96.0 mg/100 mL brewed coffee	[47]
Ferulic acid	9.1–14.3 mg/100 g coffee	[44,48,49]
*p*-Coumaric acid	Approx. 5 mg/200 mL brewed coffee (1 cup)	[50]
Sinapic acid	350–1750 mg/L coffee beverage (70–350 mg/200 mL serving); 10.34 and 10.89 mg/100 g (green/light roast beans)	[51,52]

### 3.1. Potential Role of Chlorogenic Acid in T2DM

Chlorogenic acids are abundant in green coffee beans and can be categorized into three subgroups: caffeoylquinic acids, *p*-coumaroylquinic acids, and feruloylquinic acids [53]. Each subgroup exists in multiple isomeric forms, of which 5-*O*-caffeoylquinic acid, commonly referred to as chlorogenic acid, is the predominant compound in green coffee. Chlorogenic acid constitutes approximately 76–84% of the total chlorogenic acid content in green coffee beans, with a concentration of approximately 100 mg/g dry matter, as determined by HPLC [46]. The content of total chlorogenic acids, particularly chlorogenic acid, was significantly influenced by roasting conditions. Higher roasting temperatures and intensities are correlated with a reduction in the chlorogenic acid concentration due to thermal decomposition, highlighting the impact of processing methods on the phytochemical composition of coffee beans [46,54].

Both in vitro and in vivo studies have demonstrated that chlorogenic acid exerts a wide range of pharmacological activities, including antioxidant, anti-inflammatory, antibacterial, antiviral, hypoglycemic, lipid-lowering, cardioprotective, antimutagenic, and anticancer effects [55]. Potential therapeutic effects in T2DM have also been reported [56,57,58].

#### 3.1.1. In Vitro Studies

Chlorogenic acid inhibits the key enzymes involved in carbohydrate metabolism, such as α-amylase and maltase, thereby potentially reducing postprandial glucose levels [59]. In this study, chlorogenic acid was tested at concentrations of 0–5 mM and showed inhibition of human salivary α-amylase and rat intestinal maltase activities under physiological pH conditions. Supporting this, chlorogenic acid inhibits porcine pancreatic α-amylase (PPA) isozymes (PPA-I and PPA-II) in vitro, with IC₅₀ values ranging from 0.07 to 0.08 mM [60], which may delay starch hydrolysis and contribute to lower postprandial glucose levels. In addition, chlorogenic acid has been reported to markedly inhibit Na-dependent d-glucose transport in brush border membrane vesicles in the rat small intestine [61]. Pretreatment with chlorogenic acid (1 mM) reduced active glucose uptake by approximately 80%, likely through dissipation of the Na + electrochemical gradient that drives glucose cotransport, without affecting passive glucose diffusion or membrane integrity. This finding supports the direct role of chlorogenic acid in limiting intestinal glucose absorption contributes to its glycemia-lowering potential.

Chlorogenic acid enhances glucose uptake by adipocytes [62], and in rat skeletal muscle cells in the presence of insulin [63]. It also stimulates insulin secretion from INS-1E β-cells and isolated rat pancreatic islets, suggesting a dual role in improving both insulin sensitivity and pancreatic β-cell function. A recent study has further identified protein kinase B (AKT) as a direct target of chlorogenic acid. Using chlorogenic acid-modified magnetic microspheres and immunofluorescence imaging, chlorogenic acid was shown to bind specifically to the pleckstrin homology domain of AKT, thereby inducing its phosphorylation at Ser-473 and activating downstream signaling molecules such as glycogen synthase kinase 3β and forkhead box protein O1 (FOXO1) [64]. This direct activation of the AKT pathway resulted in enhanced glucose metabolism in insulin-stimulated HepG2 cells, suggesting an insulin-sensitizing mechanism for chlorogenic acid at the molecular level.

#### 3.1.2. Animal Studies

The in vitro findings were supported by animal studies. For instance, chlorogenic acid improves glucose tolerance, suppresses hepatic glucose-6-phosphatase (G6Pase) activity, and enhances glucose uptake in the skeletal muscles of *db*/*db* mice, ultimately leading to a reduction in fasting blood glucose and an improvement in insulin sensitivity [56,65]. Mechanistically, Ong et al. [65] reported that chlorogenic acid stimulates glucose uptake in skeletal muscles via the activation of adenosine monophosphate-activated protein kinase (AMPK). In a subsequent study, the same group confirmed that chlorogenic acid reduced hepatic G6Pase expression and activity, while promoting skeletal muscle glucose uptake, resulting in improved glucose homeostasis in *db*/*db* mice [56]. These metabolic benefits are closely associated with AMPK activation, as pharmacological inhibition or knockdown of AMPK abolished the glucose-lowering effects of chlorogenic acid, indicating a central role for AMPK in mediating its anti-diabetic effects. In addition to downregulating G6Pase activity, chlorogenic acid inhibits glucose-6-phosphate hydrolysis by selectively binding to the T1 transporter, which facilitates substrate entry into the G6Pase system, without directly inhibiting the catalytic enzyme [66]. This interaction limits substrate availability, thereby reducing the overall enzyme activity.

Supporting this mechanism, a chlorogenic acid-rich instant coffee extract and its major component, chlorogenic acid, were also found to inhibit G6Pase activity in rat liver microsomes [67]. In late-stage diabetic *db*/*db* mice, chlorogenic acid administration reduced the fasting plasma glucose and hemoglobin A1c (HbA1c) levels, while improving kidney fibrosis markers [57]. These effects were associated with increased adiponectin receptor expression and activation of AMPK and peroxisome proliferator-activated receptor (PPAR)-α signaling pathways in the liver and muscle. Similar glucose-lowering effects of chlorogenic acid were also observed in an HFD-induced insulin-resistant mouse model that mimicked early-stage T2DM [58]. In this model, chlorogenic acid treatment improved insulin sensitivity and reduced fasting blood glucose levels. These metabolic improvements were accompanied by the downregulation of hepatic monoacylglycerol acyltransferase 1 (*MGAT1*), a gene implicated in insulin resistance and hepatic glucose metabolism [68], further supporting its role in improving glucose homeostasis under insulin-resistant conditions.

#### 3.1.3. Human Studies

Clinical trials have provided supporting evidence for these mechanisms in humans. Johnston et al. [69] reported that chlorogenic acid in coffee might delay glucose absorption and contribute to the modulation of postprandial insulin secretion, as indicated by increased GLP-1 levels in healthy subjects. Another randomized crossover study conducted by van Dijk et al. [70] demonstrated that acute ingestion of chlorogenic acid or trigonelline reduced early postprandial glucose and insulin levels during the oral glucose tolerance test in overweight men. Collectively, these findings suggest that chlorogenic acid may exert beneficial effects on postprandial glycemic regulation in humans. Consistently, a previous study on a 12-week intervention in patients with impaired glucose tolerance showed that supplementation with chlorogenic acid (400 mg, three times daily) significantly reduced fasting plasma glucose and insulin secretion, while improving insulin sensitivity, lipid profiles, and anthropometric measures [71]. Similarly, consumption of instant coffee enriched with chlorogenic acid was shown to reduce postprandial glucose absorption by 6.9% compared to control coffee in healthy individuals, and to significantly decrease body weight in overweight subjects over 12 weeks, suggesting the potential benefits of chlorogenic acid in regulating dietary glucose utilization and body composition [31].

However, the clinical evidence remains inconsistent. A recent RCT involving patients with T2DM and non-alcoholic fatty liver disease found that 6-month supplementation with chlorogenic acid (200 mg/day) and/or caffeine (200 mg/day) did not improve fasting glucose, homeostasis model assessment-estimated insulin resistance (HOMA-IR), HbA1c, C-peptide, inflammatory markers (e.g., TNF-α, high-sensitivity C-reactive protein), hepatic fat accumulation, fibrosis, and other pathological features [72]. Although most metabolic and hepatic outcomes did not improve, significantly lower total cholesterol levels in the caffeine group and an increase in insulin levels in the chlorogenic acid + caffeine group suggested potential context-dependent benefits.

A mechanistic interpretation was proposed to support these preclinical and clinical findings. McCarty [73] hypothesized that chlorogenic acid may suppress intestinal glucose absorption and enhance GLP-1 secretion, thereby promoting the expression of IDX-1, a transcription factor critical for maintaining β-cell responsiveness to glucose [74], and ultimately improving insulin secretory function. Furthermore, chlorogenic acid has been suggested to inhibit glucose-6-phosphate translocase 1, a key component of intestinal glucose transport, thereby delaying glucose absorption and mitigating postprandial hyperglycemia. By reducing glucose toxicity, this mechanism may contribute to the preservation of β-cell function in insulin-resistant individuals. This hypothesis suggests a plausible link between habitual coffee consumption and reduced incidence of T2DM, as observed in epidemiological studies.

Despite these mechanistic insights and promising preclinical findings, the clinical outcomes remain inconsistent. Although several preclinical and mechanistic studies have demonstrated the potential benefits of chlorogenic acid in glucose metabolism, insulin sensitivity, and hepatic lipid regulation, the results from human trials have been mixed. Acute and short-term studies in healthy and overweight individuals have reported favorable effects on postprandial glucose levels and insulin secretion, and even modest reductions in body weight. Similarly, a 12-week RCT in patients with impaired glucose tolerance showed improvements in fasting glucose levels, insulin sensitivity, lipid profiles, and anthropometric markers after high-dose chlorogenic acid supplementation. However, these findings contrast with those of a more recent 6-month RCT involving patients with T2DM and non-alcoholic fatty liver disease, in which supplementation with chlorogenic acid and/or caffeine failed to significantly improve hepatic fat content, liver stiffness, or glycemic and inflammatory markers. This discrepancy may reflect differences in the study populations (e.g., early vs. advanced metabolic disease), chlorogenic acid dose and formulation, intervention duration, or background dietary patterns. These inconsistencies underscore the need for more rigorously designed, long-term clinical trials with well-characterized chlorogenic acid formulations and stratified patient groups to clarify its therapeutic potential in human metabolic disorders.

**Figure 3:**
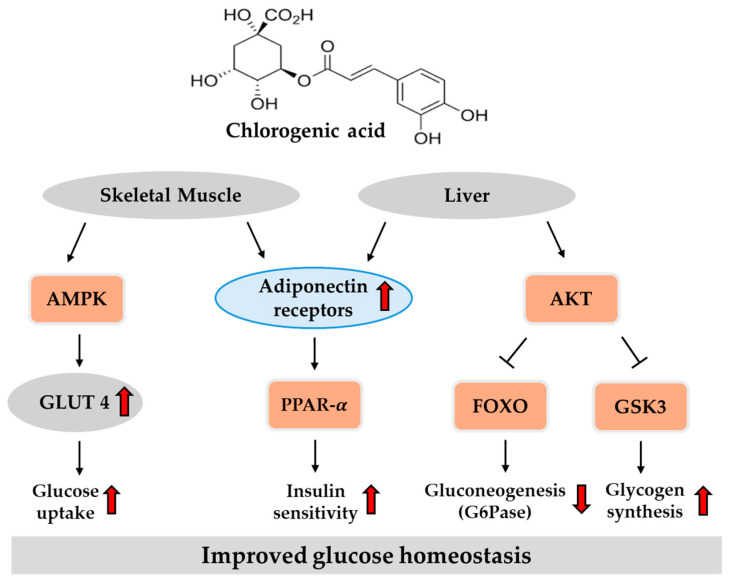
Potential pathways of chlorogenic acid in regulating glucose homeostasis in the liver and skeletal muscle. Red arrows indicate direction: ↑ increase, ↓ decrease. AMPK, adenosine monophosphate-activated protein kinase; FOXO, forkhead box protein O; GLUT, glucose transporter; GSK3, glycogen synthase kinase 3β; PPAR-α, peroxisome proliferator-activated receptor-α.

Together, evidence from in vitro and animal studies demonstrates the potential of chlorogenic acid to improve glucose metabolism through inhibition of digestive enzymes, stimulation of insulin secretion, and suppression of hepatic glucose production. Figure 3 provides an overview of the key regulatory pathways modulated by chlorogenic acid in the liver and peripheral tissue, based on previously reported findings. Although clinical outcomes have been inconsistent, they suggest possible benefits in postprandial glycemic control, emphasizing the need for further validation through well-controlled, long-term trials.

### 3.2. Potential Role of Caffeic Acid in T2DM

The caffeic acid content in brewed coffee ranges from 63.1 to 96.0 mg per 100 mL, as determined by HPLC [47]. Caffeic acid has been reported to possess various pharmacological properties, including antioxidant, anti-inflammatory, and anti-diabetic effects [75,76].

#### 3.2.1. In Vitro Studies

Caffeic acid improves insulin resistance by promoting insulin receptor tyrosyl phosphorylation, upregulating the expression of insulin signaling-associated proteins such as insulin receptors, phosphatidylinositol 3-kinase (PI3K), glycogen synthase, and glucose transporter (GLUT) 2, thereby enhancing glucose uptake in TNF-α-induced insulin-resistant FL83B hepatocytes [77]. Similarly, a recent study has shown that caffeic acid modulates the PI3K-AKT signaling pathway, contributing to its hypoglycemic effects [78]. Complementary molecular docking studies demonstrated robust binding affinities between caffeic acid and key proteins implicated in T2DM pathophysiology, namely PIK3CA, α-glucosidase, and AKT1, supporting its potential for therapeutic modulation at the intracellular signaling level.

Moreover, in INS-1E β-cells, caffeic acid enhanced glucose-stimulated insulin secretion and glucose sensitivity [79]. It has also been shown to inhibit carbohydrate-digesting enzymes such as α-amylase and β-glucosidase [80], and to suppress intestinal glucose absorption via inhibition of sodium-dependent GLUT 1 in Caco-2 cells [81]. Further studies reinforced the inhibitory effects of caffeic acid on α-amylase and α-glucosidase based on both enzymatic assays and molecular docking analyses [82,83]. Notably, McMillan et al. [83] demonstrated that caffeic acid exhibits inhibition constants (Ki) comparable to those of acarbose, a clinically prescribed enzyme inhibitor, without significant cytotoxicity in Caco-2 cells, highlighting its potential to delay carbohydrate digestion and mitigate postprandial hyperglycemia.

In addition, caffeic acid exhibits antioxidant effects, including radical scavenging, inhibition of lipid peroxidation, and prevention of glutathione depletion in hepatocytes [47]. It also promotes glucose uptake in L6 myotubes and isolated rat skeletal muscle tissue, and enhances glycogenesis while inhibiting gluconeogenesis in TNF-α-treated insulin-resistant mouse hepatocytes [84,85].

#### 3.2.2. In Vivo Studies

The anti-diabetic potential of caffeic acid has been supported by in vivo studies. In *db*/*db* mice with T2DM, caffeic acid improved glucose metabolism by upregulating pancreatic insulin expression, enhancing hepatic glucokinase activity, inhibiting hepatic gluconeogenic G6Pase and phosphoenolpyruvate carboxykinase (PEPCK) activities, and increasing GLUT4 expression in the adipose tissue, leading to reductions in fasting blood glucose and HbA1c levels [86]. Similarly, caffeic acid, a compound found in the fruit of *Xanthium strumarium*, alleviated glucose intolerance and insulin resistance in obese (*fa*/*fa*) Zucker rats [87]. Tsuda et al. [88] reported that caffeic acid promoted AMPK activity and insulin-independent glucose transport in rat skeletal muscles. In a rat model of HFD-induced hyperinsulinemia, daily oral administration of caffeic acid (30 mg/kg) for 30 weeks significantly reduced plasma glucose and insulin levels, improved insulin resistance, and upregulated insulin signaling proteins, including the insulin receptor, PI3K, AKT, and insulin-degrading enzymes in the brain [89]. In a rat model of fructose/streptozotocin (STZ)-induced T2DM, caffeic acid improved glycemic control, restored pancreatic β-cell function, and ameliorated dyslipidemia, hepatic and renal dysfunction, and oxidative stress, with efficacy comparable to that of metformin [90]. To enhance its therapeutic efficacy, recent studies have explored complexation strategies, such as zinc(II)–caffeic acid complexation, which demonstrated improved anti-diabetic and antioxidant activities in type 2 diabetic animal models compared to either component alone [91].

In addition to its metabolic actions, caffeic acid exerts vascular protective effects through multiple mechanisms. It inhibits the formation of advanced glycation end products (AGEs) and attenuates AGE-induced oxidative stress and inflammation, as evidenced by the downregulation of IL-1β, IL-18, intercellular adhesion molecule-1, vascular cell adhesion protein-1, NLRP3, caspase-1, and C-reactive protein, and by the reduction of reactive oxygen species (ROS) generation in human umbilical vein endothelial cells [92]. Moreover, caffeic acid mitigates hyperglycemia-induced endothelial dysfunction by suppressing nuclear factor kappa B (NF-κB) nuclear translocation, reducing the expression of endothelial adhesion molecules, and enhancing antioxidant defenses via nuclear factor erythroid 2-related factor (Nrf)-2/electrophile-responsive element activation [93]. It also alleviates glycated low-density lipoprotein (LDL)-induced endothelial inflammation by inhibiting AGE receptor expression, reducing oxidative and endoplasmic reticulum stress, and decreasing inflammatory mediator secretion [94]. Consistent with these effects, caffeic acid counteracted hyperglycemia-induced endothelial apoptosis by preserving barrier integrity, suppressing NF-κB signaling, downregulating caspases, and enhancing Bcl-2 phosphorylation [95].

**Figure 4:**
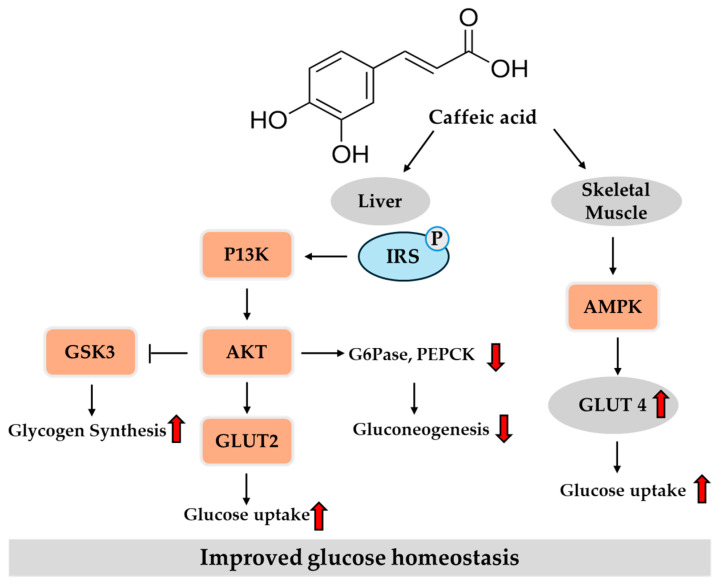
Potential pathways of caffeic acid in regulating glucose homeostasis in the liver and skeletal muscle. Red arrows indicate direction: ↑ increase, ↓ decrease. AMPK, adenosine monophosphate-activated protein kinase; GLUT, glucose transporter; GSK3, glycogen synthase kinase 3β; IRS, insulin receptor substrate; P, phospho; PI3K, phosphatidylinositol 3-kinase.

Collectively, in vitro studies suggest the potential of caffeic acid to improve insulin signaling, inhibit carbohydrate-digesting enzymes, enhance insulin secretion, promote glucose uptake, and reduce oxidative stress. In vivo findings further highlight that caffeic acid confers metabolic and vascular protection against T2DM through a multi-targeted approach involving the modulation of glucose metabolism, oxidative stress, inflammation, and glycation-related damage. Together, these in vitro and in vivo studies support its therapeutic promise in managing hyperglycemia and insulin resistance. Figure 4 provides an overview of the key regulatory pathways modulated by caffeic acid in the liver and peripheral tissue, based on previously reported findings.

### 3.3. Potential Role of Ferulic Acid in T2DM

Coffee is one of the dietary sources of ferulic acid. Studies have reported that 100 g of coffee contains approximately 9.1 to 14.3 mg of ferulic acid, as determined by HPLC [44,48,49]. Given that a standard cup of coffee is brewed using approximately 10 g of beans, this translates to an estimated 2.3 to 12 mg of ferulic acid per cup. Ferulic acid has a relatively longer plasma presence and higher bioavailability than other dietary flavonoids and monophenols [96]. After absorption, approximately 56.1% of ferulic acid enters enterocytes, where it is rapidly conjugated, and the resulting conjugates are subsequently transported to the serosal side. Approximately 6% is excreted in bile, whereas the majority (~49.9%) reaches peripheral tissues, where it is believed to exert biological effects [97]. Coffee consumption has been shown to increase plasma ferulic acid concentrations owing to the gastrointestinal metabolism of chlorogenic acid, highlighting ferulic acid as a bioavailable dietary compound with potential metabolic benefits [98]. Given these properties, ferulic acid has attracted considerable interest as a potential agent for the prevention and management of T2DM and its complications, including diabetic retinopathy, nephropathy, neuropathy, and cardiomyopathy [99,100,101].

#### 3.3.1. In Vitro Studies

Various in vitro studies have elucidated multiple mechanisms by which ferulic acid may exert anti-diabetic effects. Notably, it stimulates insulin secretion in pancreatic β-cells. Ruamyod et al. [102] reported increased insulin release in rat insulinoma cells via the activation of L-type Ca^2^⁺ channels. Similarly, Nomura et al. [103] found that amide derivatives of ferulic acid promoted insulin secretion in rat pancreatic β-cells. In addition to its pancreatic effects, ferulic acid has been shown to enhance glucose uptake and insulin signaling in peripheral tissues. A zinc (II)–ferulic acid complex strengthens these effects by increasing glucose uptake, hexokinase activity, and phospho-AKT levels in muscle cells [104]. Moreover, ferulic acid derived from *Hibiscus mutabilis* mitigates fatty acid-induced insulin resistance by restoring insulin receptor expression, possibly via the upregulation of insulin receptor substrate-1 (IRS)-1 [105,106]. Ferulic acid has also been reported to inhibit carbohydrate-digesting enzymes such as α-glucosidase and α-amylase, thereby contributing to improved postprandial glycemic control [107].

Along with its metabolic actions, ferulic acid exhibits notable cytoprotective properties and metabolic actions. It attenuates oxidative stress-induced cellular damage, as evidenced by a reduction in malondialdehyde levels and the restoration of glutathione levels in isolated pancreatic tissues [90]. In human ARPE-19 cells, ferulic acid reduces glucose-induced apoptosis, suggesting its role in preventing diabetic retinopathy [108]. Furthermore, in human erythrocytes exposed to high glucose levels, ferulic acid reduces lipid peroxidation, prevents phosphatidylserine externalization, and inhibits glycated hemoglobin formation [109].

#### 3.3.2. Animal Studies

In vivo studies further support the anti-diabetic potential of ferulic acid. In several diabetic animal models, ferulic acid ameliorated oxidative stress markers, enhanced antioxidant enzyme activities, and preserved pancreatic and vascular tissue integrity [100,101,110,111]. These protective effects were mediated through the suppression of ROS generation, inhibition of aldose reductase activity, and modulation of key signaling pathways including PI3K/AKT, NF-κB, p38 MAPK, and TGF-β/Smad [110]. Anti-inflammatory, anti-fibrotic, and endothelial-protective actions have also been reported. For example, ferulic acid administration (150–300 mg/kg/day) in rats with fructose/STZ-induced diabetes significantly reduced blood glucose levels, increased serum insulin concentrations, enhanced antioxidant defenses (e.g., glutathione, superoxide dismutase (SOD), and catalase), and preserved pancreatic morphology and β-cell ultrastructure, as evidenced by histological and transmission electron microscopy analyses [111]. Similar protective effects have also been observed in rats with STZ-induced diabetes, in which ferulic acid reduced blood glucose, thiobarbituric acid-reactive substances, hydroperoxides, and free fatty acid levels, while enhancing antioxidant enzyme activities and preserving pancreatic islet architecture [111].

Ferulic acid consistently improved glycemic control and insulin sensitivity in multiple models of T2DM, including *KK-Ay* mice, *db*/*db* mice, and HFD/STZ-induced insulin-resistant animals [112,113,114,115]. In *db*/*db* mice, ferulic acid (50 mg/kg for 17 days) improved hyperglycemia by increasing hepatic glucokinase activity and glycogen storage [114]. Ferulic acid (50 mg/kg for 30 days) also regulated hepatic glucose metabolism in rats with HFD- and fructose diet-induced T2DM [116]. Accordingly, it enhanced glycogen synthesis and suppressed gluconeogenesis, as indicated by increased hepatic glycogen content, upregulated glucokinase and glycogen synthase activities, and reduced PEPCK and G6Pase expression through the inhibition of FOXO1 promoter binding, with effects comparable to those of metformin. The same research group also demonstrated that ferulic acid reduced *GLUT2* expression by modulating transcription factors such as sterol regulatory element-binding protein 1c (SREBP1c), hepatocyte nuclear factor *(HNF)1α*, and *HNF3β* [117]. Specifically, ferulic acid was shown to downregulate the expression of *HNF1α* and *HNF3β*, both of which are positive regulators of *GLUT2* gene transcription, and to suppress SREBP1c activity, thereby attenuating the transcriptional activation of *GLUT2*. This coordinated regulation likely contributes to the reduction in hepatic glucose output under diabetic conditions. In rats treated with HFD and low-dose STZ, long-term administration of ferulic acid (60 mg/kg for 16 weeks) reduced circulating glucose, lipids, and tissue damage markers (alanine transaminase, aspartate transaminase, creatine kinase, and lactate dehydrogenase), while attenuating hepatic and cardiac apoptosis and upregulating antioxidant genes such as heme oxygenase-1 and glutathione S-transferase [115].

In addition to its effects as a single agent, ferulic acid has been studied as a multi-component phytochemical formulation. In a rat model of low-dose STZ/HFD-induced diabetes, a mixture of gymnemic acid, trigonelline, and ferulic acid significantly improved glucose homeostasis, suppressed oxidative stress and inflammation, and preserved pancreatic histology, suggesting possible synergistic effects [118]. Furthermore, feruloylated oligosaccharides derived from maize bran showed greater anti-diabetic efficacy than free ferulic acid in rats with low-dose STZ/HFD-induced diabetes [119]. At a dose of 600 mg/kg/day, these oligosaccharides significantly reduced the levels of fasting glucose, insulin, triglycerides, LDL cholesterol, and tissue injury markers (aspartate transaminase, creatine kinase, and lactate dehydrogenase), while increasing HDL cholesterol levels and inhibiting AGE formation in multiple organs.

A maternal supplementation study using HFD- and fructose diet-fed rats reported that ferulic acid (50 mg/kg/day) prevented hyperglycemia, pancreatic islet inflammation, and developmental retardations in offspring [120]. Its protective role in pancreatic islet inflammation was accompanied by reduced NF-κB activation. These findings suggest that ferulic acid administration in mothers with T2DM provides beneficial effects on the offspring, likely through its direct protective action on pancreatic β-cells.

In addition to its antioxidant, anti-inflammatory, and β-cell-protective effects, emerging evidence suggests that ferulic acid may exert anti-diabetic effects by modulating the gut microbiome. In rats with low-dose STZ/HFD-induced diabetes, ferulic acid altered the gut microbial composition by increasing beneficial genera, such as *Bacteroides*, *Blautia*, *Faecalibacterium*, and *Parabacteroides*, while decreasing potentially harmful taxa [121]. It also increased the production of short- and branched-chain fatty acids which are microbial metabolites known to influence host glucose metabolism. These findings highlight gut microbiota modulation as an additional mechanism contributing to the therapeutic potential of ferulic acid.

In combination therapy, ferulic acid combined with metformin has been reported to exert synergistic effects in both in vitro and in vivo models [122]. Combination therapy improves glucose uptake and glycemic control more effectively than either agent alone, allowing for a four-fold reduction in the required metformin dose without compromising therapeutic efficacy. This synergy is attributed to the complementary activation of the PI3K pathway by ferulic acid and the AMPK pathway by metformin, both of which converge to regulate glucose uptake. Importantly, ferulic acid has no adverse effects, even at high doses or in combination, and co-administration is associated with an increased number of pancreatic islets [123].

#### 3.3.3. Human Studies

Supporting its in vivo antioxidant effects, a clinical trial has shown that ferulic acid-rich coffee increases plasma antioxidant capacity. Following coffee intake, elevated plasma levels of ferulic acid have been correlated with an improved antioxidant status in humans [124]. However, a randomized dietary intervention study in obese individuals reported that ferulic acid-enriched cereal products improved oxidative stress markers, but did not significantly affect fasting or postprandial glucose, lipid profiles, or inflammatory markers [125]. These findings reveal a discrepancy between the promising preclinical outcomes and the relatively modest effects observed in clinical trials.

Therefore, further clinical research should be conducted to clarify the therapeutic potential of ferulic acid for glycemic control. In particular, well-designed RCTs should be performed in the future to determine optimal dosing, treatment duration, and formulation strategies for enhancing bioavailability. Identifying patient subgroups that may benefit the most from ferulic acid and evaluating its use in combination with existing anti-diabetic agents may yield more clinically meaningful outcomes. A comprehensive assessment of insulin sensitivity, β-cell function, glycemic variability, and conventional metabolic parameters is essential to fully establish the clinical relevance of ferulic acid in the management of T2DM.

**Figure 5:**
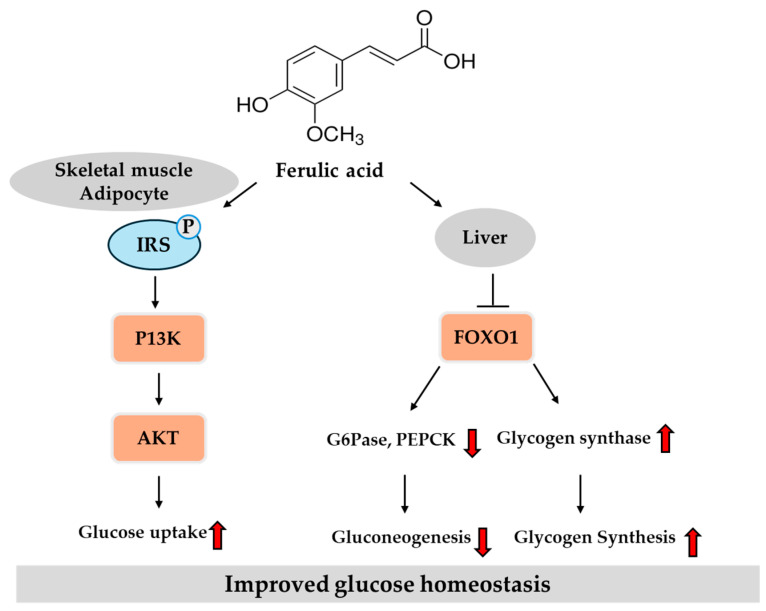
Potential pathways of ferulic acid in regulating glucose homeostasis in the liver and peripheral tissues. Red arrows indicate direction: ↑ increase, ↓ decrease. FOXO, forkhead box protein O; G6Pase, glucose-6-phosphatase; IRS, insulin receptor substrate; P, phospho; PEPCK, phosphoenolpyruvate carboxykinase; PI3K, phosphatidylinositol 3-kinase.

Collectively, in vitro studies demonstrate that ferulic acid enhances insulin secretion, improves glucose uptake and insulin signaling, inhibits carbohydrate-digesting enzymes, and exerts cytoprotective and antioxidant effects. In vivo evidence further supports its potential to ameliorate hyperglycemia and insulin resistance by reducing oxidative stress, inflammation, and tissue damage, preserving pancreatic and vascular integrity, and modulating key metabolic signaling pathways. Additionally, the ability of ferulic acid to favorably alter gut microbiota and act synergistically with metformin highlights its multifaceted therapeutic potential. Clinical studies have confirmed its antioxidant benefits but revealed inconsistent effects on glycemic control, underscoring the need for further well-designed trials to optimize dosing, formulations, and target populations. Figure 5 provides an overview of the key regulatory pathways modulated by ferulic acid in liver and peripheral tissues, based on previously reported findings.

### 3.4. Potential Role of p-Coumaric Acid in T2DM

*p*-Coumaric acid is a naturally occurring hydroxycinnamic acid found in fruits, vegetables, cereals, and coffee. Green coffee beans contain various *p*-coumaric-acid-containing chlorogenic acids, and *p*-coumaric acid has been detected in brewed coffee [126]. The concentration of *p*-coumaric acid in a typical cup of brewed coffee (~200 mL) has been reported to be around 5 mg, as quantified by HPLC [50], although the concentration can vary depending on factors such as the coffee species, bean processing, and brewing methods [126]. *p*-Coumaric acid is structurally related to ferulic acid and has several biological activities, including antioxidant, anti-inflammatory, and metabolic regulatory effects. Because of these properties, *p*-coumaric acid has garnered increasing attention for its potential therapeutic role in the prevention and management of T2DM.

#### 3.4.1. In Vitro Studies

In vitro studies have provided mechanistic insights into the anti-diabetic actions of *p*-coumaric acid. Previous studies reported that *p*-coumaric acid inhibited α-amylase activity in vitro [127] and exhibited appreciable binding affinity toward α-amylase, as confirmed by fluorescence quenching and thermodynamic analyses [128]. Consistent with these findings, a recent study demonstrated that *p*-coumaric acid exhibited α-amylase and α-glucosidase inhibitory activities comparable to those of acarbose, suggesting its potential to attenuate postprandial hyperglycemia via digestive enzyme inhibition [83]. Furthermore, *p*-coumaric acid has been shown to inhibit AGE formation to a lesser extent than caffeic acid, likely because of its lower hydroxyl content. These findings highlight the multi-targeted anti-diabetic potential of *p*-coumaric acid via both digestive enzyme inhibition and antiglycation pathways. Inhibiting AGE formation is particularly important, because AGEs contribute to the pathogenesis of diabetic complications such as nephropathy and retinopathy [129]. Moreover, *p*-coumaric acid has been shown to activate AMPK in various cells (skeletal muscle cells, adipocytes, and hepatocytes), which enhances lipid oxidation and improves insulin signaling [130,131,132].

#### 3.4.2. In Vivo Studies

*p*-Coumaric acid exerts anti-diabetic effects, partly through its antioxidant capacity which aids in mitigating oxidative stress, a central contributor to pancreatic β-cell dysfunction and insulin resistance [133]. While the direct evidence regarding β-cell protection is limited, *p*-coumaric acid (100 mg/kg body weight) administration has been demonstrated by animal studies to lower blood glucose levels, increase circulating insulin levels, and restore the level of glutathione, a key non-enzymatic antioxidant, in rats with STZ-induced diabetes [134]. Furthermore, histopathological findings indicate that *p*-coumaric acid (100 mg/kg body weight) protects the pancreas, liver, and kidneys and helps in maintaining both enzymatic and non-enzymatic antioxidant activity, supporting its potential role in preserving tissue integrity and alleviating oxidative damage under diabetic conditions [135]. One previous report revealed that, at a dose of 20 mg/kg body weight, it also reduced oxidative stress, upregulated antioxidant enzyme activity, and improved kidney function biomarkers in rats with HFD-induced diabetes, suggesting a renoprotective effect against diabetic nephropathy [136].

In addition to its antioxidant and tissue-protective effects, *p*-coumaric acid (0.02%, *w*/*w*) may act through metabolic regulatory pathways that contribute to its anti-diabetic effects. For example, it has been shown to suppress hepatic gluconeogenesis by inhibiting key enzymes such as G6Pase and PEPCK, thereby reducing blood glucose levels in high-fructose-diet-fed hamsters [137]. At a dietary concentration of 0.002% (*w*/*w*), it also improved glucose homeostasis and insulin sensitivity in HFD-fed mice [138].

Moreover, recent evidence suggests that *p*-coumaric acid exerts multi-organ protective effects in mice with HFD/STZ-induced T2DM by enhancing intestinal nutrient absorption, preserving intestinal barrier function, and activating hepatic IRS-1/PI3K/AKT signaling, thereby further improving systemic glucose metabolism [139]. Additionally, at a dose of 200 mg/kg body weight, it modulates hypothalamic leptin signaling and promotes whole-body glucose homeostasis in both cellular and animal models, potentially through the differential regulation of the AMPK pathway [140].

In addition to diet-induced models of insulin resistance, *p*-coumaric acid (2 mg/mL in drinking water) shows anti-diabetic efficacy in *db*/*db* mice, a widely used genetic model of T2DM [141]. In this model, *p*-coumaric acid improved fasting blood glucose levels, glucose tolerance, and insulin sensitivity. Although the primary focus of this study was to enhance thermogenesis via the mTORC1–RPS6 signaling pathway in brown adipose tissue, the results also revealed a significant increase in the oxidation of both fatty acids and glucose. This enhanced uptake and glucose utilization in brown adipose tissue was associated with the increased expression of glucose transporters (GLUT1, GLUT3, and GLUT4) and the glycolytic enzyme pyruvate kinase-R, suggesting a direct role of *p*-coumaric acid in promoting glucose clearance and improving systemic glucose homeostasis.

Consistent with these findings, another in vivo study using a rat model of nicotinamide- and STZ-induced T2DM demonstrated that oral *p*-coumaric acid administration (40 mg/kg body weight) significantly improved glycemic control, enhanced insulin secretion, and increased body weight while reducing glucose and HbA1c levels [142]. Moreover, *p*-coumaric acid modulated key adipocytokines by decreasing TNF-α levels, increasing adiponectin secretion, and upregulating *PPAR-γ* mRNA expression, which contributed to improvements in lipid profiles, cardiovascular risk indices, and anti-atherogenic status. These results further support the notion that *p*-coumaric acid exerts its anti-diabetic effects not only through antioxidant and metabolic pathways but also via direct modulation of adipose tissue function and insulin sensitization mechanisms.

**Figure 6:**
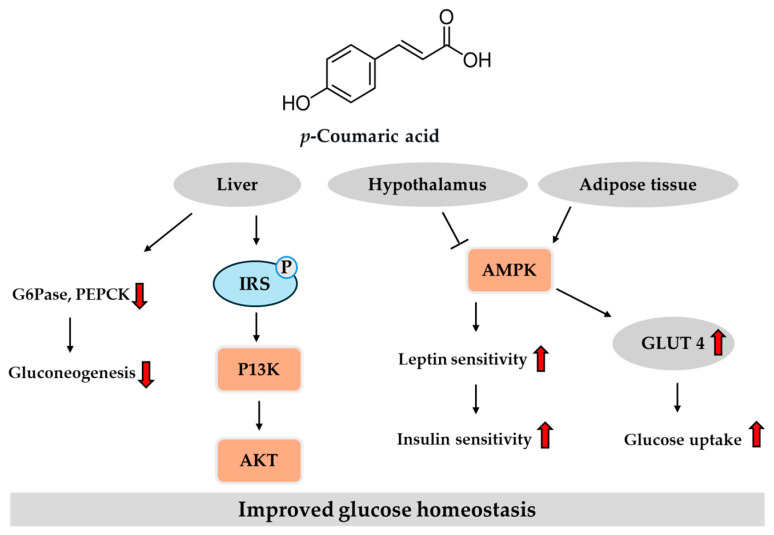
Potential pathways of *p*-coumaric acid in regulating glucose homeostasis in the liver, hypothalamus and adipose tissue. Red arrows indicate direction: ↑ increase, ↓ decrease. AMPK, adenosine monophosphate-activated protein kinase; GLUT, glucose transporter; G6Pase, glucose-6-phosphatase; IRS, insulin receptor substrate; P, phospho; PEPCK, phosphoenolpyruvate carboxykinase; PI3K, phosphatidylino-sitol 3-kinase.

These in vitro and in vivo studies suggest that *p*-coumaric acid may exert anti-diabetic effects through multiple mechanisms, including the inhibition of digestive enzymes, antioxidant activity, and modulation of metabolic signaling pathways and adipocytokine secretion. Figure 6 provides an overview of the key regulatory pathways modulated by *p*-coumaric acid in the liver, hypothalamus and adipose tissue, based on previously reported findings. Despite these promising preclinical findings, human studies directly evaluating the anti-diabetic efficacy of *p*-coumaric acid remain limited. Given its natural occurrence in commonly consumed foods such as coffee and whole grains, along with its demonstrated safety profile in animal models, *p*-coumaric acid is a promising candidate for future clinical research. Well-designed human trials are warranted to assess its bioavailability, optimal dosage, and therapeutic efficacy when administered either independently or alongside currently available anti-diabetic treatments.

### 3.5. Potential Role of Sinapic Acid in T2DM

Sinapic acid, a hydroxycinnamic acid present in coffee beans, has variable concentrations depending on the coffee species, processing, and brewing conditions [143]. As estimated by Manach et al. [51], coffee beverages contain approximately 350–1750 mg of sinapic acid per liter, corresponding to an intake of 70–350 mg per standard serving. A study by Somporn et al. [52], based on HPLC analysis, reported that the sinapic acid content of Arabica coffee beans slightly increased following light roasting (10.89 mg/100 g) compared to green beans (10.34 mg/100 g), but declined substantially with higher roasting levels, reaching 2.88 mg/100 g and 5.27 mg/100 g in medium and dark roasts, respectively. These findings suggest that sinapic acid levels may initially increase during light roasting, but substantially decline under higher thermal conditions due to degradation.

Both in vitro and in vivo studies have identified multiple mechanisms by which sinapic acid may exert anti-diabetic effects. One such mechanism involves the enhancement of glucose uptake. Sinapic acid has been shown to increase insulin-independent glucose uptake in the soleus muscle isolated from rats with STZ-induced diabetes, as well as in L6 skeletal muscle cells cultured under high-glucose conditions [144]. Another mechanism involves modulation of glucose-regulating enzymes. In an HFD/low-dose STZ-induced T2DM animal model, the oral administration of sinapic acid (25 mg/kg/day for 30 days) significantly improved glycemic control, as indicated by reductions in fasting blood glucose and glycosylated hemoglobin levels, along with increased plasma insulin levels [145]. It restores the activity of key hepatic enzymes involved in glucose metabolism by upregulating glucokinase, pyruvate kinase, and glucose-6-phosphate dehydrogenase, while downregulating G6Pase and fructose-1,6-bisphosphatase. Collectively, these enzymatic changes contribute to improved glucose homeostasis. Another study using a rat model of HFD/low-dose STZ-induced diabetes further confirmed the anti-diabetic potential of sinapic acid [146]. Oral administration of sinapic acid (25 mg/kg/day for 30 days) restored hepatic glycogen levels and normalized glycogen synthase and glycogen phosphorylase activities, further supporting its role in hepatic glucose storage and utilization.

In addition to its beneficial effects on glucose metabolism, sinapic acid exerts protective effects against oxidative stress, which is a critical factor contributing to diabetic complications, such as cardiovascular disease. Zych et al. [147] demonstrated that the oral administration of sinapic acid (25 mg/kg/day for 28 days) significantly ameliorated hyperglycemia and oxidative stress in the cardiac tissue and serum of female rats with HFD/STZ-induced T2DM, as indicated by enhanced activities of key antioxidant enzymes, including SOD, catalase, and glutathione peroxidase. These findings suggest that sinapic acid may mitigate cardiovascular complications by enhancing antioxidant defenses, thus offering multifaceted therapeutic potential for diabetes management.

Further evidence supporting the therapeutic potential of sinapic acid in T2DM has been derived from studies examining diabetic nephropathy, a common complication associated with chronic hyperglycemia and insulin resistance. In an animal model of STZ-induced diabetic nephropathy, administration of sinapic acid (20 and 40 mg/kg body weight) improved renal function parameters, including reduced levels of blood urea nitrogen, serum creatinine, and urinary protein excretion [148]. These beneficial effects were associated with attenuation of oxidative stress and inflammation, as demonstrated by increased expression of the antioxidant defense proteins Nrf-2, heme oxygenase-1, and anti-apoptotic Bcl-2, coupled with reduced expression of pro-inflammatory NF-κB, TNF-α, IL-6, and pro-apoptotic Bax. Therefore, sinapic acid is a potential adjunctive therapeutic agent for the management of T2DM and its associated complications.

**Figure 7:**
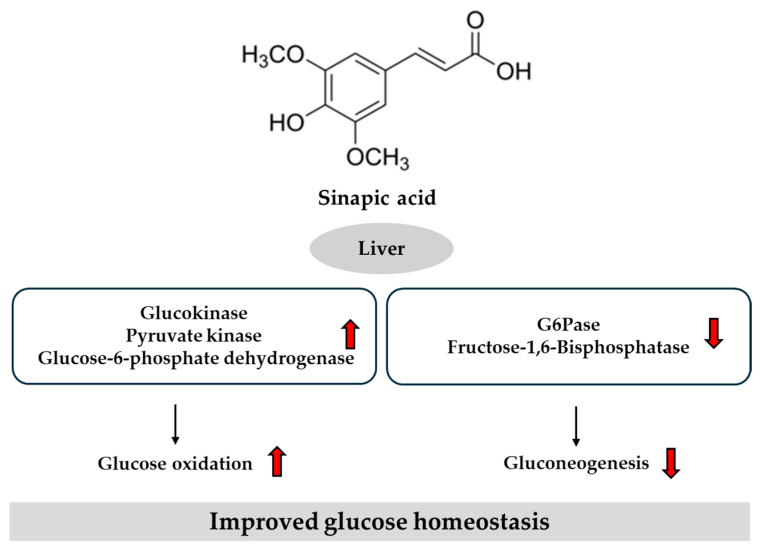
Potential pathways of sinapic acid in regulating glucose homeostasis in the liver. Red arrows indicate direction: ↑ increase, ↓ decrease. G6Pase, glucose-6-phosphatase.

Together, these in vitro and in vivo studies demonstrate the multifaceted anti-diabetic mechanisms of sinapic acid. It enhances glucose uptake through insulin-independent pathways and modulates key hepatic enzymes involved in glucose metabolism. Additionally, it restores hepatic glycogen levels and regulates enzymes associated with glycogen storage and utilization. Sinapic acid also exerts protective effects against oxidative stress by enhancing the activity of endogenous antioxidant enzymes. Its nephroprotective effects are supported by improvements in renal function markers and modulation of oxidative stress and pro-inflammatory signaling pathways. Figure 7 provides an overview of the key regulatory pathways modulated by sinapic acid in the liver, based on previously reported findings.

Despite these promising preclinical findings, careful consideration of the potential drug interactions is necessary. A recent pharmacokinetic study reported that sinapic acid co-administration significantly altered the pharmacokinetics of ertugliflozin, an SGLT2 inhibitor, by inhibiting cytochrome P450 enzymes, thereby increasing ertugliflozin exposure [149]. Thus, dose adjustments of the co-administered drugs may be necessary to avoid adverse effects.

## 4. Limitations and Future Directions

While substantial preclinical evidence supports the anti-diabetic potential of chlorogenic acid and its related hydroxycinnamic acids, several limitations must be acknowledged. Most available studies have been conducted in vitro or using animal models, which may not fully replicate the complex pathophysiology of human T2DM. The variability in experimental models, dosages, treatment durations, and compound formulations further complicates the interpretation and generalization of the findings. Nevertheless, these preclinical studies provide critical insights into underlying mechanisms and serve as an essential foundation for guiding future clinical research. Therefore, based on these preclinical findings, well-designed clinical trials are necessary to confirm their efficacy and safety in humans. Addressing these gaps will help clarify their roles as functional food components or adjunctive therapies in the management of T2DM.

Moreover, the bioavailability of these phenolic compounds is relatively low and subject to significant inter-individual variability, largely influenced by the gut microbiota composition, enzymatic metabolism, and dietary context. Potential interactions with standard anti-diabetic medications, as observed with sinapic acid and ertugliflozin, raise additional concerns that require careful pharmacokinetic and pharmacodynamic evaluations.

Future clinical investigations should focus on well-designed RCTs to establish the efficacy, optimal dosing, and long-term safety profiles of these compounds in diverse patient populations. Moreover, advanced molecular approaches, such as transcriptomic and proteomic analyses, are warranted to elucidate their specific molecular targets, signaling pathways, and gene expression profiles. These studies could provide a deeper mechanistic understanding of their anti-diabetic actions and help identify biomarkers predictive of response. In addition, studies exploring advanced delivery systems to enhance bioavailability, the synergistic effects with existing anti-diabetic therapies, and the identification of patient subgroups most likely to benefit are critical for translating these promising findings into clinical practice.

## 5. Conclusions

Coffee and its bioactive compounds, including chlorogenic acid and its hydroxycinnamic acid derivatives (namely caffeic, ferulic, *p*-coumaric, and sinapic acids) have significant potential in the prevention and management of T2DM. Preclinical studies have consistently demonstrated their beneficial effects of these compounds on glucose homeostasis, insulin resistance, oxidative stress, and inflammation, underscoring their therapeutic potential. Nevertheless, while epidemiological and mechanistic evidence suggests a protective association between coffee consumption and a reduced risk of T2DM, further clinical research is necessary to validate these findings in humans. Well-designed RCTs are needed to establish optimal dosing strategies, assess long-term safety, and clarify the bioavailability issues associated with these compounds. In addition, personalized approaches that consider genetic variability, gut microbiome composition, and individual metabolic responses may optimize the therapeutic application of coffee-derived polyphenols. Future research should continue to elucidate the multifaceted mechanisms by which chlorogenic acid and its hydroxycinnamic acid derivatives regulate metabolic health and explore their integration into comprehensive strategies for the prevention and management of T2DM.

## Figures and Tables

**Table 1:** Overview of human studies on the impact of coffee on T2DM.

Study	Population/Model	Key Findings
Huxley et al. [15]	457,000+ participants (18 prospective cohorts; median follow-up: 2–20 yrs)	7% ↓ risk per cup/day (dose-dependent)
Bhupathiraju et al. [16]	120,000+ participants (3 U.S. cohorts; follow-up: 4 yr diabetes risk assessed after 4 yr coffee intake)	12% ↓ risk with >1 cup/day
Iso et al. [17]	Japanese adults (follow-up: 5 yrs)	42% ↓ risk with ≥3 cups/day
Muley et al. [18]	1,200,000+ participants (13 cohorts; varied follow-up: 5–18 yrs	↓ risk with 4–6 or ≥6–7 cups/day (filtered and decaf. favored)
Ding et al. [19]	1,100,000+ participants (28 prospective cohorts; follow-up: 10 mos~18 yrs)	33% ↓ risk with 6 cups/day (dose-dependent)
Jiang et al. [20]	1,000,000+ participants (meta-analysis; varied follow-up: 2.6–24 yrs)	21–30% ↓ risk in highest vs. lowest intake (coffee, decaf., caf.)
van Dam et al. [26]	Healthy adults (4 wk intervention)	↑ fasting insulin (caf. coffee)/no change in glucose
Wedick et al. [27]	Overweight adults (8 wk intervention)	↑ adiponectin (caf.), ↓ fetuin-A (decaf.)
Kempf et al. [30]	Nondiabetic adults < 65 y at elevated risk of T2DM (8 wk intervention)	↑ CGA/CA metabolites, ↓ IL-18, ↑ adiponectin, HDL C with 8 cups/day
Thom [31]	Healthy adults/Overweight adults (12 wk intervention)	6.9% ↓ glucose absorption in healthy adults/5.4 kg ↓ body weight in overweight adults (CGA-enriched coffee)
Ohnaka et al. [32]	Overweight men with IFG (16 wk intervention)	↓ post-load glucose and waist (caf.); slight ↓ glucose (decaf.)
Fernández-Cardero et al. [33]	Overweight adults (12 wk intervention)	↓ fat % (LRC > RC); ↑ muscle %; no weight or MetS change

Arrows indicate direction: ↑ increase, ↓ decrease. decaf.: decaffeinated; caf.: caffeinated; T2DM: type 2 diabetes mellitus; CGA: chlorogenic acid; CA: caffeic acid; HDL C: high-density lipoprotein cholesterol; IFG: impaired fasting glucose; LRC: lightly roasted coffee; RC: roasted coffee; MetS: metabolic syndrome.

## Abbreviations

The following abbreviations are used in this manuscript:

AGEs	Advanced glycation end products
AMPK	Adenosine monophosphate-activated protein kinase
FOXO1	Forkhead box protein O1
G6Pase	Glucose-6-phosphatase
GLP-1	Glucagon-like peptide-1
GLUT2	Glucose transporter 2
HbA1c	Hemoglobin A1c
HDL	High-density lipoprotein
HFD	High-fat diet
HNF	Hepatocyte nuclear factor
HPLC	High-performance liquid chromatography
IL	Interleukin
LDL	Low-density lipoprotein
MGAT1	Monoacylglycerol acyltransferase 1
NF-κB	Nuclear factor kappa B
Nrf	Nuclear factor erythroid 2-related factor
PEPCK	Phosphoenolpyruvate carboxykinase
PI3K	Phosphatidylinositol 3-kinase
PPA	Pancreatic α-amylase
PPAR	Peroxisome proliferator-activated receptor
RCTs	Randomized controlled trials
ROS	Reactive oxygen species
SGLT2	Sodium glucose cotransporter 2
SOD	Superoxide dismutase
SREBP1c	Sterol regulatory element-binding protein 1c
STZ	Streptozotocin
TNF-α	Tumor necrosis factor-α
T2DM	Type 2 diabetes mellitus
